# An Identification of Functional Genetic Variants in *B4GALNT2* Gene and Their Association with Growth Traits in Goats

**DOI:** 10.3390/genes15030330

**Published:** 2024-03-03

**Authors:** Liang Xu, Zitong Chen, Shuheng Chen, Yu Chen, Jiazhong Guo, Tao Zhong, Linjie Wang, Siyuan Zhan, Li Li, Hongping Zhang, Jiaxue Cao

**Affiliations:** 1 Key Laboratory of Livestock and Poultry Multiomics, Ministry of Agriculture and Rural Affairs, College of Animal Science and Technology, Sichuan Agricultural University, Chengdu 611130, China; xl18383535205@163.com (L.X.); chenzt6@163.com (Z.C.); shuheng.chen@hotmail.com (S.C.); jiazhong.guo@sicau.edu.cn (J.G.); zhongtao@sicau.edu.cn (T.Z.); wanglinjie@sicau.edu.cn (L.W.); lily@sicau.edu.cn (L.L.); zhp@sicau.edu.cn (H.Z.); 2 Xinjiang Yili Prelecture Animal Husbandry Station, Yining 835000, China; 3 Sichuan Nanjiang Yellow Goat Breeding Farm, Nanjiang 635600, China; 13881675379@163.com

**Keywords:** *B4GALNT2*, SNP, association analysis, goats, growth traits

## Abstract

β-1,4-N-acetylgalactosamine transferase 2 (*B4GALNT2*) is a vital candidate gene that affects the growth traits in sheep. However, whether it has the same function in goats remains to be investigated further. This study selected 348 Nanjiang Yellow goats, screened all exons, and conserved non-coding regions of the *B4GALNT2* gene for single-nucleotide polymorphisms (SNPs). Our results revealed the presence of a synonymous mutation, rs672215506, within the exon of the *B4GALNT2* gene in the Nanjiang Yellow goat population. The mutation resulted in a decrease in the mRNA stability of the *B4GALNT2* gene. The results of SNP detection of the conserved non-coding region of the *B4GALNT2* gene showed five potential regulatory SNPs in the Nanjiang Yellow goat population. Except for rs66095343, the ~500 bp fragments of the other four SNPs (rs649127714, rs649573228, rs652899012, and rs639183528) significantly increased the luciferase activity both in goat skeletal muscle satellite cells (MuSCs) and 293T cells. The genetic diversity indexes indicated low or intermediate levels for all six SNPs analyzed, and the genotype frequencies were in Hardy–Weinberg equilibrium. Association analysis showed that rs660965343, rs649127714, and rs649573228 significantly correlate with growth traits in the later stage of growth and development of Nanjiang Yellow goats. The haplotype combinations of H2H3 and H2H2 had higher body weight and greater body size. Moreover, H2H2 haplotype combinations significantly correlated with the litter size of the Nanjiang Yellow goats. The results of our study demonstrate the potential role of the *B4GALNT2* gene as a functional genetic marker in the breeding programs of Nanjiang Yellow goats.

## 1. Introduction

β-1,4-N-acetylgalactosamine transferase 2 (*B4GALNT2*) belongs to the family of N-acetyl galactosaminyl transferase (GalNAc-Tases), which is mainly involved in the formation of O-glycosylation [1]. O-glycosylation is intricately associated with cellular recognition, adhesion, immune response and other vital biological processes [2]. The *B4GALNT2* gene is currently under extensive investigation for its involvement in animal immunology [1,3,4], gastrointestinal disorders [5,6,7,8,9], and its association with litter size. Additionally, *B4GALNT2* has been identified as a potential candidate for the genetic regulation of *Fec^L^* mutations in sheep [10]. Furthermore, the *Fec^L^* gene and its fertile allele *Fec^LL^* are among the critical genetic factors influencing ovulation in sheep [11]. Additionally, the *B4GALNT2* gene has been implicated in congenital muscular dystrophy [12], and its expression in the muscles of mice is dynamic [13]. The manipulation of *B4GALNT2* expression can affect the expression of several modifiers associated with muscular dystrophy, and the deletion of this gene exacerbates the severity of congenital muscular dystrophy in mice [14]. These findings suggest that *B4GALNT2* plays a role in muscle development.

The majority of investigations on the impact of *B4GALNT2* polymorphisms on animal production characteristics have primarily focused on sheep reproductive traits [15,16,17]. While *B4GALNT2* is located on chromosome 19 in goats, studies describing *B4GALNT2* variations associated with the number of lambs in Inner Mongolia White cashmere goats have been reported [18]. However, there is a lack of research investigating changes in goat *B4GALNT2* performance, particularly regarding growth parameters.

Boer goats are known for their excellent growth rate and meat production performance, and the 37,020,001~37,180,000 region of chromosome 19 contains many highly selective SNPs, among which the *B4GALNT2* gene and its non-coding region are located [19]. In our previous studies [20,21], this gene and its adjacent regions were strongly selective among Chinese native goat breeds with high reproductive performance and small body size (Meigu goat and Jianchang Black goat), which suggested that *B4GALNT2* may be a candidate gene affecting the performance and growth traits in goats as well. Consequently, we postulated that genetic variations at the *B4GALNT2* gene loci were associated with growth traits and litter size in goats.

Through this study, we aimed to establish a theoretical basis for selecting and breeding Nanjiang Yellow goats by identifying functional regions containing regulatory elements and investigating the association between these *B4GALNT2* gene variants and growth traits at different stages of development, as well as litter size.

## 2. Materials and Methods

### 2.1. Animals and Samples Collection

The Nanjiang Yellow goat population (*n* = 348) used in the experiment originated exclusively from the Nanjiang Yellow goat stock farm. All goats were subjected to identical management practices and environmental conditions throughout this study. Grazing and appropriate supplementary feeding were employed to raise the goats, ensuring their dietary nutrient levels met their growth requirements. In total, 1.5 mL of whole blood was collected from each test goat via jugular vein puncture, anticoagulated with heparin sodium, and stored at −20 °C for subsequent genomic DNA extraction. Pregnant ewes were randomly selected (*n* = 3) (unrelated) and humanely sacrificed. Different tissues (longissimus dorsi muscle (LD), lung, heart, spleen, liver, and kidney) were obtained.

### 2.2. Skeletal Muscle Satellite Cells (MuSCs) Isolation and Identification

According to previous methods, the LD muscle of the 1-day-old goat (male) was successfully used to isolate the MuSCs for this study [20]. Then, we used the antibody against myogenic marker genes Pax7 (Santa Cruz, CA, USA) and MyHC (Santa Cruz, CA, USA) for immunofluorescence. We stored MuSCs in liquid nitrogen tanks. The identification results are shown in Figure S1.

### 2.3. Cell Culture and Transfection

MuSCs were cultured at 5% CO_2_ and 37 °C in growth medium containing 89% Dulbecco’s modified eagle medium (DMEM), 10% fetal bovine serum (FBS; Gibco, NY, USA), and 1% penicillin-streptomycin (Invitrogen, NY, USA) [21]. Plasmids were transfected into MuSCs using Lipofectamine 3000 (Life Technologies, Carlsbad, CA, USA).

### 2.4. Plasmid Construction and RNA Stability Assays

The CDS sequences of the *B4GALNT2* (NM_001314262.1) gene were amplified with specific primers, and the full length of CDS was inserted into the pEGFP-N1 (Promega, WI, USA) vector using a homologous recombinant cloning kit (Vazyme, Nanjing, China) to construct overexpression plasmids. A site-directed mutagenesis kit (Vazyme, Nanjing, China) was used to obtain mutant sequences, and the vector was constructed in the same way. Primers were designed via single-fragment cloning (vazyme.com (1 May 2022)) CE Design and are listed in Tables S5 and S6.

Actinomycin D (AcTD, A1410, Sigma-Aldrich, St. Louis, MO, USA) was used on *B4GALNT2-G* or *B4GALNT2-G* MuSCs for 0 h, 1 h, 2 h, 4 h, and 6 h to inhibit global mRNA transcription [22].

### 2.5. Total RNA Isolation and qPCR

Total RNA was isolated from tissues and MuSCs using RNAiso Plus (Takara, Dalian, China). The cDNAs were obtained using the PrimeScript™ RT kit (Takara, Dalian, China). In addition, SYBR Premix Ex TaqTM II (Takara, Dalian, China) was used for qPCR. GAPDH was used as a reference gene and the 2^−ΔΔCt^ method was applied to normalize relative RNA expression. Primers are shown in Table S3.

### 2.6. Luciferase Reporter Assays

The fragments containing five single-nucleotide polymorphism (SNP) sites (rs660965343, rs649127714, rs639183528, rs652899012 and rs649573228) were separately inserted into the pGL3-promoter vector (Knp I and Xho I were restriction sites). Wild-type (WT) and mutation-type (MUT) plasmids were transfected into H293T and MuSCs, respectively. The dual-luciferase reporter kit (Transgen, Beijing, China) was used to detect luciferase activity. Primers used for restricting enzyme digestion are shown in Table S7.

### 2.7. Extraction of Genomic DNA and Detection of DNA Quality

Goat genomic DNA was extracted with a routine blood genome extraction kit (Tiangen, Beijing, China) and then subjected to 1.5% agarose gel electrophoresis and ultraviolet imaging in gel image analyzer BIO-RAD ChemDOC XRS. The images were analyzed using Quality One 4.6.2 software to determine DNA integrity. The purity and concentration of DNA were determined using a nucleic acid protein detector (BIO-RAD, Hercules, CA, USA). The samples that met the requirements were stored at −20 °C for later use. Gel electrophoresis is shown in Figure S2.

### 2.8. PCR Amplification and Sequencing

Based on the SNP position, each SNP and its flanking sequences were retrieved from the Ensembl database, and primers were designed using the sequence as a template using Primer Premier 5.0 software and were synthesized by Sangon (Shanghai, China). The birth record table of each goat in the Nanjiang Yellow goat breeding farm was consulted, and 20 DNA samples were selected and diluted to a concentration of 20 ng/μL. From each sample, 2 μL of DNA was extracted and thoroughly mixed. The resulting mixed pool of DNA served as the template for PCR amplification. The PCR products were sent to Shanghai Sangon (Shanghai, China) for bidirectional Sanger sequencing. SnapGene6.0.2 software was used to verify the SNPS in the samples by comparing the sequencing results with the reference genome sequence and SNP sites. Primers are shown in Table S4.

### 2.9. MassARRAY Genotyping

In total, 348 Nanjiang Yellow goats were genotyped using the Sequenom MassARRAY genotyping technique. According to the information of 6 SNP sites in the DNA samples of 348 Nanjiang Yellow goats, SNP sites and the information of 100 bp upstream and downstream sequences were obtained through the Ensembl database. Subsequently, we amplified the fragments containing SNP sites with the single-base primer extension method, combined with MALDI-TOF, and distinguished genotypes according to their molecular weight. The blood genomic DNA of all samples was submitted to Fuyu Biotechnology (Beijing, China) for genotyping.

### 2.10. Growth Trait Determination

The birth weight, body weight (BW), body length (BL), body height (BH), and chest circumference (CC) of Nanjiang Yellow goats (*n* = 348) were measured using standard methods at the ages of 6 months, 12 months, and 18 months. Birth weight: weight taken within 12 h of birth; BW: body weight measured three times using the steelyard to take the average; BL: straight line distance from the leading edge of the scapula to the hip; BH: the vertical distance from the highest point of the girth to the ground; CC: the length around the chest from the back end of the shoulder blade. The primary data on the growth development and reproductive performance of Nanjiang Yellow goats are shown in Table S9.

### 2.11. Bioinformatics Analysis and Data Analysis

Jaspar (http://jaspar.genereg.net/ (accessed on 2 March 2022)) was used to predict changes in transcription factor binding at mutation sites in non-coding regions.

Haploview4.2 was used to calculate Hardy–Weinberg equilibrium and analyze linkage disequilibrium among SNPs. PHASE 2.1.1 software was used to construct haplotypes. SAS 9.4 software was used to analyze the association between genotypes of each locus and the growth traits of Nanjiang Yellow goats, and the GLM model in SAS 9.4 was used to establish the model. Y_ijkl_ = μ + G_i_ + S_j_ +P_k_ + D_l_ + e_ijkl_, where Y_ijkl_ represented the phenotypic observations; μ was the averaged values; G_i_ was the fixed effect of genotype; S_j_ was the fixed effect of sex; P_k_ was the fixed effect of place; D_l_ was the fixed effect of date of birth (year and month); and e_ijkl_ was the random effect. All values were expressed as mean ± standard deviation. The results with *p* < 0.05 were considered statistically significant. 

## 3. Results

### 3.1. The Synonymous Mutation rs672215506 Affected the mRNA Stability of B4GALNT2 Gene

**Figure 1:**
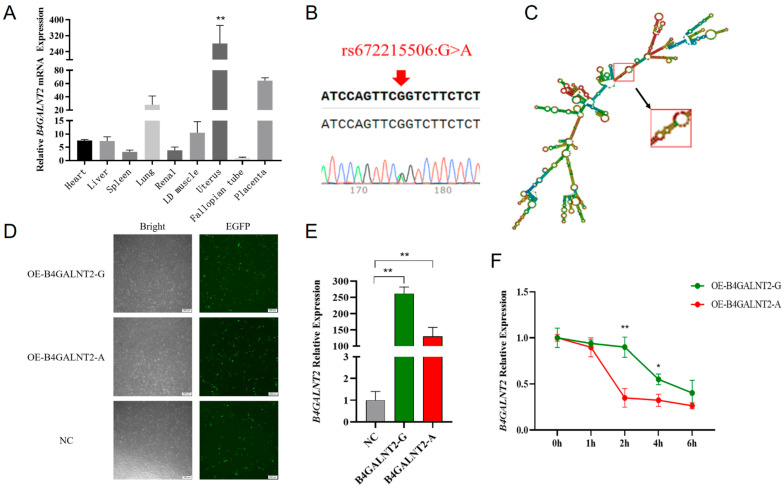
The expression pattern of the *B4GALNT2* gene in various tissues and the effects of *B4GALNT2* synonymous mutation on mRNA stability. (**A**) *B4GALNT2* gene expression profile in different tissues of goats. (**B**) SNPs sequencing peak of *B4GALNT2* exons. The base indicated by the arrow in the figure represents the genomic locus of the corresponding SNP. A double peak indicates the presence of this SNP within the Nanjiang Yellow goat population. (**C**) Prediction of the secondary structure of *B4GALNT2* mRNA. Only those in the red boxes were altered, and the arrows represent the mutated mRNA secondary structure. (**D**) The transfection efficiencies of wild-type and mutant-type overexpression vectors in goat MuSCs were assessed. Cells carrying green fluorescence represent successful transfection. (**E**) The expression levels of the G and A genotype overexpression vectors of the *B4GALNT2* (*B4GALNT2-G* and *B4GALNT2-A*) were detected in goat MuSCs. (**F**) The relative expression levels of the wild type and mutant type of the *B4GALNT2* gene in goat MuSCs were evaluated after treatment with actinomycin D for different times. Results are represented as mean ± SEM, * *p* < 0.05, ** *p* < 0.01.

We performed a qPCR to explore the expression profile of *B4GALNT2* in goat tissues. (Figure 1A). Notably, a significantly higher expression level was observed in the uterus compared to other tissues (*p* < 0.01). This result suggests a potential correlation between this gene and reproductive traits in goats. Then, we screened all exonic SNPs through the utilization of the Ensembl online database and previously collected resequencing data from Nanjiang Yellow goats in our laboratory, resulting in a total discovery of 10 SNPs. Those SNPs were validated through mixed-pool PCR and sequencing in the Nanjiang Yellow goat population, resulting in the identification of only rs672215506, a synonymous mutation, in the 9 exons (Figure 1B).

To validate the function of the rs672215506 mutation, firstly, we predicted the secondary structure of *B4GALNT2* mRNA. It revealed that there was only a slight change between the wild type and mutation type, which is shown in the red box (Figure 1C). Secondly, we calculated the free energy in both types; it was observed that after the mutation, there was a slight increase in the minimum free energy from −2087.40 kJ/mol to −2078.20 kJ/mol. Lastly, we tested its mRNA stability between wild type and mutant type. After successfully transfecting the *B4GALNT2* gene, it either contained wild-type G or mutant-type A, in goat MuSCs, (Figure 1D), which was confirmed via the RT-qPCR method (Figure 1E). Moreover, we found that the expression of *B4GALNT2* mRNA was significantly reduced after the addition of ACTD at 2 h and 4 h compared to the wild type (Figure 1F) (*p* < 0.05 or *p* < 0.01).

### 3.2. The Detection and Functional Verification of Non-Coding SNPs in B4GALNT2

**Figure 2:**
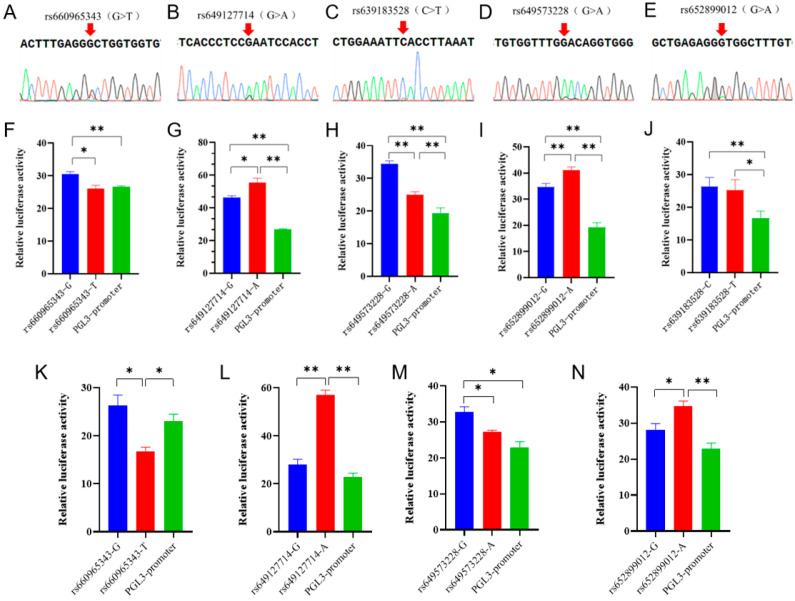
Five SNPs sequencing results and the effects of mutations on enhancer activity. (**A**–**E**) Five SNPs sequencing results of mixed pool samples, which were located at the non-coding region of *B4GALNT2* gene. The base indicated by the arrow in the figure represents the genomic locus of the corresponding SNP. A double peak indicates the presence of this SNP within the Nanjiang Yellow goat population. (**F**–**J**) Effects of mutations on enhancer activity. Blue represents the mutant type, red represents the wild type, and green represents control. They were transfected into 293T cells. (**K**–**N**) Effects of mutations on enhancer activity. They were transfected into MuSCs cells. The results are expressed as mean ± SEM (*n* = 3 or 4) in arbitrary units based on firefly luciferase activity normalized against Renilla luciferase activity. A t-test was conducted using SPSS 25.0 to detect the differences. Bars represent mean ± SEM of at least three repeats. * *p* < 0.05; ** *p* < 0.01.

In the Ensembl database, the SNPs in the non-coding region of the goat *B4GALNT2* gene were analyzed with homology analysis with 42 other species, and 5 conserved SNPs were screened (Figure S3). Sanger sequencing results showed that these SNPs were present in the Nanjiang Yellow goat population (Figure 2A–E). Then, the analysis of transcription factor binding sites associated with these SNPs was conducted via Jaspar, and numerous transcription factors were found to significantly impact the binding affinity (Table S1). To further investigate the functionality of these sites, wild-type and mutant-type dual-luciferase reporter vectors were constructed for each site and subsequently transfected into 293T cells. The results showed that the dual-luciferase activity of the wild-type group at all five sites was extremely significantly higher than the control group, indicating that the fragment containing these SNPs had enhancer-like activity (Figure 2F–J). Specifically, both rs660965343 and rs649573228 mutations led to a significant reduction in luciferase activity (*p* < 0.05 or *p* < 0.01) (Figure 2F,H), whereas rs649127714 and rs652899012 mutations resulted in a significant increase (*p* < 0.05 or *p* < 0.01) (Figure 2G,I), while rs639183528 did not show any significant changes (*p* > 0.05) (Figure 2J). Furthermore, dual-fluorescent vectors exhibiting significant disparities in 293T cells were selected and transfected into MuSCs to investigate whether these motifs possess identical functionality in muscle cells. The results showed that the luciferase activity of the wild-type and mutant-type vectors showed a similar trend in both cell types (Figure 2K–N). It is interesting that the luciferase activity of the rs660965343 mutant in muscle cells exhibited a significant reduction, even surpassing that of the PGL3-promoter (control) group (*p* < 0.05). The implication of this finding is that this mutation may play a more significant role in the functionality of muscle cells.

### 3.3. Population Genetic Diversity Statistics of Six SNPs in Nanjiang Yellow Goat Population

**Figure 3:**
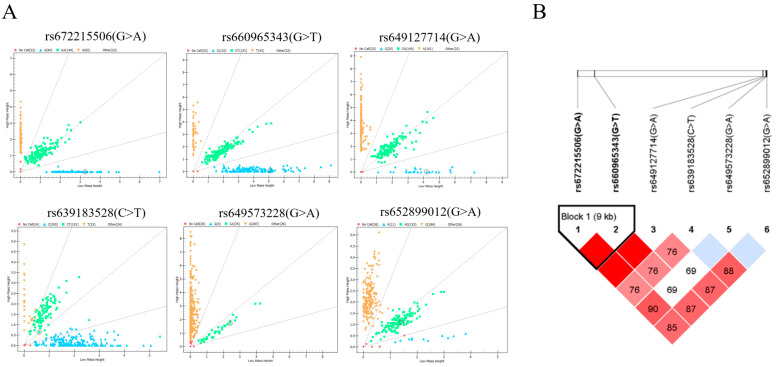
Mass spectrometry results and linkage disequilibrium analysis. (**A**) SNP genotyping results are shown as scatter plots, with different colored dots representing different genotypes. (**B**) Linkage disequilibrium analysis of six SNPs in Nanjiang Yellow goat population.

From the results above, we identified one SNP located at nine exons and five SNPs in the non-coding region, totaling six SNPs in the *B4GALNT2* genes. Then, we asked the question of whether they exist in the goats. The mass spectrometry method was applied to analyze the genetic distribution of these SNPs in 348 Nanjiang Yellow goats; the results showed that six SNPs all existed in the goat population (Figure 3A). Among them, rs652062749 had the highest homozygosity, and rs672215506 had the lowest homozygosity. The analysis of effective allele numbers for different SNPs revealed that the overall effective allele number is approximately 2, except for rs652062749, which shows relatively low values, indicating an even distribution of effective alleles within the population. The genetic statistics of the goat population showed that all mutations were moderately or weakly polymorphic in the Nanjiang Yellow goats (0.25 < PIC ≤ 0.5 or PIC ≤ 0.25). The Chi-squared fitness test showed that the SNP distribution was in Hardy–Weinberg equilibrium (*p* > 0.05, Table 1).

### 3.4. Analysis of Linkage Disequilibrium and Construction of Haplotypes

The presence of SNPs in linkage disequilibrium can provide additional insights into genetic information, and it was conducted based on the SNPs and genotyping information (Figure 3B). A strong linkage disequilibrium was observed between rs672215506 and rs660965343 (D′ = 1.000). Subsequently, two highly linked SNPs were used to construct three haplotypes, with the GG haplotype having the highest frequency (0.487). Additionally, six different combinations of these haplotypes were identified. Except for H1H1, all other haplotype combinations had frequencies higher than 5% (Table 2).

### 3.5. Association of SNPs and Haplotype Combinations with Growth Traits

To validate these SNPs’ function in vivo, we analyzed their association with growth traits both in a SNP locus and haplotype combination. For a single SNPs’ part, we found that the birth weight of those with the AA genotype was significantly higher than that of goats with the GG genotype in the rs672215506 site, (*p* < 0.05) (Table 3). However, the BH-6, BL-12, and BH-12 measurements in goats with the AA genotype were significantly lower compared to those with the GG genotype (*p* < 0.05 or *p* < 0.01). Individuals with the TT genotype at rs660965343 exhibited a significant advantage in terms of birth weight (*p* < 0.05), but demonstrated inferiority during subsequent periods (*p* < 0.05 or *p* < 0.01). The goats with the AA genotype of rs649127714 exhibited significantly larger body size or heavier body mass compared to goats with other genotypes at all periods (*p* < 0.05 or *p* < 0.01). The individuals with the CC genotype of rs639183528 exhibited significantly higher BL-6 levels compared to the TT genotype (*p* < 0.05). The rs649573228 locus did not exhibit any GG homozygous genotype. In goats with the AA genotype, BL, BH-6, CC-12, BL-12, BH-12, BL-18, BH-18, and CC-18 showed significantly better results compared to the GA genotype (*p* < 0.05 or *p* < 0.01). The rs652899012 AA genotype exhibited a significantly lower frequency compared to the AG genotype in BL-6, BH-6, CC-6, BL-12, and BH-12 (*p* < 0.05 or *p* < 0.01) (Table 4). For the haplotype combinations part, overall, H3H3 exhibited inferior performance in terms of body weight and body size compared to other haplotype combinations (*p* < 0.05 or *p* < 0.01, Table 5)

### 3.6. Association of SNPs and Haplotype Combinations with Lambing Number

The *B4GALNT2* gene was well known for affecting lamb number in sheep, which was never tested in goat population—to the best of our best knowledge. Then, we investigated the association between six SNPs and lambing performance in primiparous and multiparous Nanjiang Yellow goat populations (Table S2). Even though the results showed no significant variations in lambing performance among different genotypes of each SNP in primiparous and multiparous ewes (*p* > 0.05), and no significant correlations between individual haplotype combinations and the number of primiparous lambs (*p* > 0.05), interestingly, the H2H2 haplotype combination exhibited a significantly higher multiparous lamb count than the other haplotype combinations (*p* < 0.05, Table 6).

## 4. Discussion

*B4GALNT2*, highly expressed in sheep’s ovaries [17], has received attention as a critical candidate gene affecting lamb birth and ovulation rate [22,23,24]. The *B4GALNT2* protein of goats has close homology with that of sheep (Figure S4), and it has been reported that *B4GALNT2* mRNA is expressed in the ovaries, uteri, and fallopian tubes of goats [25]. Consistent with this, we found that the expression of *B4GALNT2* was highest in the uteri of the Nanjiang Yellow goats. These results suggest that *B4GALNT2* may also be critical for goats, but this gene has been poorly studied in goats.

The *B4GALNT2* gene and its non-coding region 37,020,001–37,180,000 on chromosome 19 of Boer goats have strong selection signals [19], and they are also strongly selected in small Chinese local goat breeds (Meigu goats and Jianchang goats) [26,27]. This suggests that this gene is closely related to the growth and development of goats. However, goats’ variations in the *B4GALNT2* gene, especially those associated with growth traits, have not been extensively studied yet.

In this study, we identified six SNPs in the *B4GALNT2* gene located in the conserved non-coding region and exons of the gene in 348 Nanjiang Yellow goats (known for their fast growth and high reproductive efficiency in China). We found significant associations between these SNPs and production traits and their number of lambs in Nanjiang Yellow goats, which provide insights for the further characterization of the production performance of livestock.

Synonymous mutations occurring in exons do not alter amino acid or protein sequences. However, they may regulate gene function by affecting codon bias during protein translation [28,29,30], and studies have shown that synonymous mutations can affect animal production performance by influencing the efficiency of transcription and translation of genes [31,32,33], for example, a synonym mutation on the IGF1 gene in Bama pigs altered the stability of *IGF1* mRNA and protein [33]. In goats, a recent study found that GG and GA genotypes with a synonym mutation (g.37072289 G>A) in the *B4GALNT2* gene had significantly higher lamb births than other genotypes [18]. In this study, a synonymous mutation rs672215506 (G>A) was identified in the *B4GALNT2* gene of goats. Moreover, the birth weight of the AA genotype was significantly higher than that of the GG genotype. We also found that this synonym mutation decreased the mRNA stability of *B4GALNT2* after a mutation, which might be why this site affected the birth weight of the Nanjiang Yellow goats.

SNPs in non-coding regions can indirectly regulate gene expression processes, thereby affecting animal phenotypes or reproductive performance [34,35]. The dual-luciferase reporter vector assay is a precise and dependable method for validating non-coding SNPs in research [36,37,38]. In this study, we carried out a dual-luciferase assay in two cell types, 293T and MuSCs. Interestingly, the rs660965343 mutant-type vector showed lower luciferase activity in MuSCs. This locus may have unique effects on muscle development, but further studies are needed.

The level of genetic variation within a population is the most direct expression of genetic diversity, and the level of genetic variation can directly affect the evolutionary potential of the population [39,40]. Population heterozygosity is an essential indicator for judging the genetic diversity of a certain population, which can reflect the degree of genetic diversity of a population [41]. All six SNPS in this study were in Hardy–Weinberg equilibrium, and there was rich genetic diversity within the Nanjiang Yellow goat population, which had good purification and selection potential [42]. In addition, all loci had low (PIC ≤ 0.25) or moderate polymorphism (0.25 < PIC ≤ 0.5), with some genetic variation potential [43,44].

The influence of genes on traits may be influenced by the linkage effect of multiple SNPs [45,46]. The linkage disequilibrium of SNPs can provide more comprehensive genetic information and enhance selection efficiency [47,48]. In this study, the haplotype combination of H2H3 and H2H2 was found to be beneficial for increasing body weight and size, while H2H2 showed advantages for increasing the number of multiparous lambs. In addition, the mean value of the multiparous lambing number was significantly higher than the number of primiparous lambing numbers, which was in line with the results of a previous study [49].

## 5. Conclusions

In conclusion, we identified five SNPs of *B4GALNT2* which are strongly associated with growth traits in goats. Our findings contribute to a better understanding of the genetic mechanisms underlying growth traits in Nanjiang Yellow goats and emphasize the importance of the *B4GALNT2* gene in breeding strategies aimed at enhancing productivity and performance in this economically valuable breed.

## Figures and Tables

**Table 1:** Genetic parameters and Hardy–Weinberg equilibrium test of six SNPs in the goat population.

Locus	Genotype	Genotype Frequency	Allele Frequency	Ho	He	Ne	*p*	PIC
rs672215506 (G>A)	AA	0.28	0.51 (A)	0.5	0.5	2	0.16	0.37
	GA	0.46	0.49 (G)					
	GG	0.26						
rs660965343 (G>T)	GG	0.4	0.64 (G)	0.54	0.46	1.85	1	0.35
	GT	0.46	0.36 (T)					
	TT	0.14						
rs649127714 (G>A)	GG	0.08	0.29 (G)	0.59	0.41	1.7	0.58	0.33
	GA	0.43	0.71 (A)					
	AA	0.49						
rs639183528 (C>T)	CC	0.62	0.77 (C)	0.65	0.35	1.54	0.07	0.29
	CT	0.31	0.23 (T)					
	TT	0.07						
rs649573228 (G>A)	AA	0.89	0.95 (A)	0.9	0.1	1.11	0.75	0.1
	GA	0.11	0.05 (G)					
rs652899012 (G>A)	GG	0.58		0.65	0.35	1.54	0.09	0.29
	AG	0.39	0.77 (G)					
	AA	0.03	0.23 (A)					

Note: Ho, He, Ne, and PIC represent homozygosity, heterozygosity, effective number of alleles, and polymorphism information content, respectively; *p* > 0.05 indicates the locus was under Hardy–Weinberg equilibrium.

**Table 2:** Haplotype and haplotype combination construction.

Haplotype and Haplotype Combination	Type	Genotype	Frequency
Haplotype	H1	GG	0.487
	H2	AG	0.141
	H3	AT	0.372
	H1H3	GGAT	0.379
Haplotype combination	H2H2	GGGG	0.241
	H3H3	ATAT	0.124
	H2H3	AGAT	0.118
	H1H2	GGAG	0.112
	H1H1	AGAG	0.026

**Table 3:** Association analysis of six SNPs with birth weight in Nanjiang Yellow goat population.

Locus	Genotype	Number	Birth Weight
rs672215506 (G>A)	AA	92	2.29 ± 0.28 ^a^
	GA	149	2.26 ± 0.29 ^ab^
	GG	84	2.20 ± 0.31 ^b^
rs660965343 (G>T)	TT	43	2.29 ± 0.26 ^a^
	GT	151	2.27 ± 0.33 ^ab^
	GG	132	2.22 ± 0.27 ^b^
rs649127714 (G>A)	AA	161	2.27 ± 0.34
	GA	140	2.24 ± 0.24
	GG	25	2.22 ± 0.30
rs639183528 (C>T)	CC	200	2.26 ± 0.32
	CT	101	2.24 ± 0.26
	TT	23	2.23 ± 0.25
rs649573228 (G>A)	AA	287	2.25 ± 0.31
	GA	35	2.24 ± 0.24
	AA	11	2.26 ± 0.26
rs652899012 (G>A)	AG	125	2.22 ± 0.27
	GG	186	2.27 ± 0.32

Note: Different small letters in the same group mean significant difference (*p* < 0.05).

**Table 4:** Association analysis of six SNPs with various body size traits for different amounts of months in the Nanjiang Yellow goat population.

Locus	rs672215506 (G>A)			rs660965343 (G>T)			rs649127714 (G>A)		
Genotype	AA	GA	GG	TT	GT	GG	AA	GA	GG
Number	60	88	49	26	89	82	100	82	15
BW-6	25.99 ± 3.87	25.83 ± 4.45	26.28 ± 4.58	25.42 ± 3.67 ^b^	25.99 ± 4.16 ^ab^	26.17 ± 4.64 ^a^	26.66 ± 4.42 ^A^	25.26 ± 3.91 ^B^	25.53 ± 5.03 ^B^
BL-6	58.85 ± 5.21	59.06 ± 5.42	59.73 ± 5.53	58.00 ± 4.43 ^B^	59.16 ± 5.23 ^A^	59.54 ± 5.79 ^A^	59.79 ± 5.59 ^A^	58.37 ± 4.98 ^B^	59.33 ± 5.77 ^AB^
BH-6	55.80 ± 4.44 ^B^	56.11 ± 4.96 ^A^	56.11 ± 4.96 ^A^	55.23 ± 3.92 ^B^	56.21 ± 4.79 ^A^	56.43 ± 4.80 ^A^	56.71 ± 4.99 ^A^	55.50 ± 4.24 ^B^	56.27 ± 4.67 ^AB^
CC-6	65.19 ± 4.02	65.06 ± 4.56	65.71 ± 4.83	64.37 ± 3.65 ^B^	65.17 ± 4.23 ^AB^	65.65 ± 4.92 ^A^	65.89 ± 4.69 ^Aa^	64.54 ± 4.09 ^Bb^	65.07 ± 4.48 ^ABb^
BW-12	34.08 ± 4.54	34.22 ± 5.04	34.49 ± 5.31	33.37 ± 4.18 ^Bb^	34.37 ± 4.80 ^ABa^	34.39 ± 5.33 ^Aa^	35.02 ± 5.23 ^A^	33.38 ± 4.33 ^B^	33.83 ± 5.55 ^B^
BL-12	65.70 ± 4.76 ^Bb^	66.11 ± 5.14 ^ABb^	66.80 ± 5.65 ^Aa^	64.73 ± 4.19 ^Bc^	66.13 ± 5.01 ^Ab^	66.63 ± 5.54 ^Aa^	66.72 ± 5.46 ^A^	65.44 ± 4.68 ^B^	66.33 ± 5.39 ^AB^
BH-12	62.75 ± 4.04 ^b^	63.13 ± 4.55 ^b^	63.73 ± 4.71 ^a^	62.15 ± 3.56 ^Bb^	63.08 ± 4.37 ^ABa^	63.57 ± 4.74 ^Aa^	63.61 ± 4.69 ^a^	62.55 ± 4.03 ^b^	63.53 ± 4.70 ^a^
CC-12	74.86 ± 4.93	75.26 ± 5.28	75.87 ± 5.20	74.12 ± 4.73 ^Bb^	75.23 ± 4.92 ^ABa^	75.73 ± 5.49 ^Aa^	75.76 ± 5.51	74.76 ± 4.74	75.10 ± 4.73
BW-18	47.23 ± 6.43	48.39 ± 7.62	48.20 ± 7.45	46.33 ± 6.08 ^B^	48.20 ± 7.37 ^A^	48.29 ± 7.39 ^A^	48.67 ± 7.79 ^a^	47.10 ± 6.08 ^b^	48.30 ± 8.80 ^ab^
BL-18	72.78 ± 4.80	73.56 ± 5.42	73.53 ± 5.65	71.35 ± 4.23 ^B^	73.48 ± 5.11 ^A^	73.76 ± 5.68 ^A^	73.77 ± 5.58 ^a^	72.82 ± 4.84 ^b^	73.00 ± 5.66 ^b^
BH-18	69.17 ± 3.95	69.30 ± 4.31	69.41 ± 4.38	68.38 ± 3.42 ^B^	69.25 ± 4.22 ^AB^	69.61 ± 4.40 ^A^	69.69 ± 4.43 ^A^	68.70 ± 3.69 ^AB^	69.80 ± 5.10 ^B^
CC-18	85.52 ± 4.66	85.85 ± 5.10	85.71 ± 4.88	84.46 ± 4.34 ^B^	85.93 ± 4.62 ^A^	85.88 ± 5.33 ^A^	86.32 ± 5.13 ^a^	85.04 ± 4.37 ^b^	85.37 ± 5.80 ^b^
**Locus**	**rs639183528 (C > T)**			**rs649573228 (G>A)**			**rs652899012 (G>A)**		
Genotype	CC	CT	TT	AA		GA	AA	AG	GG
Number	119	63	15	174		21	7	71	117
BW-6	26.38 ± 4.45	25.47 ± 3.85	25.10 ± 4.69	26.14 ± 4.28		24.86 ± 4.56	25.71 ± 5.19	25.19 ± 4.00	26.46 ± 4.41
BL-6	59.45 ± 5.54 ^a^	58.89 ± 5.03 ^ab^	58.00 ± 5.57 ^b^	59.30 ± 5.41 ^A^		58.05 ± 5.31 ^B^	60.14 ± 6.15 ^A^	58.49 ± 5.09 ^B^	59.56 ± 5.52 ^AB^
BH-6	56.45 ± 4.97	55.76 ± 4.08	55.67 ± 4.82	56.32 ± 4.70 ^A^		55.14 ± 4.67 ^B^	57.29 ± 5.22 ^A^	55.48 ± 4.16 ^B^	56.53 ± 4.97 ^AB^
CC-6	65.55 ± 4.72	64.92 ± 4.07	64.40 ± 3.96	65.44 ± 4.47 ^A^		64.00 ± 4.42 ^B^	65.71 ± 4.03 ^a^	64.65 ± 4.23 ^b^	65.62 ± 4.64 ^ab^
BW-12	34.60 ± 5.17	33.79 ± 4.47	33.30 ± 5.02	34.43 ± 4.95 ^A^		32.67 ± 4.91 ^B^	34.07 ± 5.76	33.48 ± 4.48	34.71 ± 5.18
BL-12	66.39 ± 5.34	65.87 ± 4.76	65.47 ± 5.44	66.29 ± 5.22 ^A^		65.24 ± 4.82 ^B^	66.57 ± 6.16 ^a^	65.58 ± 4.72 ^b^	66.51 ± 5.39 ^ab^
BH-12	63.34 ± 4.66	62.86 ± 3.95	63.00 ± 4.77	63.29 ± 4.46 ^A^		62.24 ± 4.41 ^B^	63.86 ± 5.24 ^a^	62.65 ± 4.01 ^b^	63.43 ± 4.67 ^ab^
CC-12	75.45 ± 5.34	75.30 ± 5.02	73.97 ± 3.95	75.41 ± 5.18		74.52 ± 5.06	75.36 ± 5.02	74.80 ± 4.80	75.58 ± 5.40
BW-18	48.41 ± 7.58	47.40 ± 6.42	47.10 ± 7.66	48.14 ± 7.34		47.05 ± 6.45	47.79 ± 8.32	47.20 ± 6.63	48.53 ± 7.55
BL-18	73.61 ± 5.52	72.98 ± 4.91	72.40 ± 5.03	73.40 ± 5.32 ^a^		73.00 ± 5.2 ^b^	73.29 ± 5.88	72.77 ± 4.96	73.7 ± 5.48
BH-18	69.47 ± 4.34	68.94 ± 3.82	69.27 ± 4.76	69.4 ± 4.24 ^A^		68.57 ± 4.01 ^B^	68.71 ± 4.82	68.83 ± 3.81	69.55 ± 4.34
CC-18	86.02 ± 5.10	85.33 ± 4.53	84.87 ± 4.81	85.84 ± 4.92 ^A^		84.60 ± 4.91 ^B^	85.00 ± 4.93	85.08 ± 4.72	86.13 ± 5.03

Note: Values are shown as means ± standard deviation; ^a,b,c^ means with different superscripts are significantly different (*p* < 0.05); ^A,B^ means with different superscripts are very significantly different (*p* < 0.01); body weight for six months (BW-6), body length for six months (BL-6), body height for six months (BH-6), chest circumference for six months (CC-6), and so on.

**Table 5:** Association analysis between haplotype combination and growth traits of Nanjiang Yellow goats.

Combined Haplotypes	H2H3	H3H3	H1H2	H1H3	H2H2
Number	27	26	26	75	49
BW-6	26.30 ± 3.63 ^a^	25.42 ± 3.67 ^b^	25.77 ± 4.67 ^ab^	25.66 ± 4.42 ^ab^	26.28 ± 4.58 ^ab^
BL-6	59.52 ± 5.4 ^Aa^	58 ± 4.43 ^Bb^	59.19 ± 6.06 ^Aa^	58.8 ± 5.29 ^ABab^	59.73 ± 5.53 ^Aa^
BH-6	56.11 ± 4.46 ^Aa^	55.23 ± 3.92 ^Bb^	55.77 ± 5.04 ^ABa^	56.07 ± 5.02 ^ABa^	56.73 ± 4.51 ^Aa^
CC-6	65.70 ± 3.71 ^Aa^	64.37 ± 3.65 ^Bc^	65.35 ± 4.91 ^Aab^	64.74 ± 4.40 ^ABbc^	65.71 ± 4.83 ^Aab^
BW-12	34.33 ± 4.46 ^a^	33.37 ± 4.18 ^b^	33.83 ± 5.30 ^ab^	34.14 ± 4.95 ^ab^	34.49 ± 5.31 ^a^
BL-12	66.07 ± 4.82 ^Aab^	64.73 ± 4.19 ^Bc^	66.00 ± 5.29 ^Aab^	66.02 ± 5.09 ^Ab^	66.80 ± 5.65 ^Aa^
BH-12	62.85 ± 4.03 ^ABa^	62.15 ± 3.56 ^Bb^	63.00 ± 4.67 ^ABa^	63.01 ± 4.47 ^ABb^	63.73 ± 4.71 ^Aa^
CC-12	75.35 ± 4.90 ^Aa^	74.12 ± 4.73 ^Bb^	75.46 ± 6.05 ^Aa^	74.88 ± 4.94 ^Bb^	75.87 ± 5.20 ^Aa^
BW-18	47.76 ± 6.53 ^Aa^	46.33 ± 6.08 ^Bb^	48.38 ± 7.45 ^Aa^	47.93 ± 7.45 ^Ba^	48.20 ± 7.45 ^Aa^
BL-18	73.56 ± 4.55 ^Aab^	71.35 ± 4.23 ^Bc^	73.81 ± 5.64 ^Aa^	73.2 ± 5.22 ^Ab^	73.53 ± 5.65 ^Aab^
BH-18	69.52 ± 4.05 ^Aab^	68.38 ± 3.42 ^Bc^	69.69 ± 4.34 ^Aa^	69.03 ± 4.16 ^Ab^	69.41 ± 4.38 ^Aab^
CC-18	86.33 ± 4.07 ^Aa^	84.46 ± 4.34 ^Bb^	86.08 ± 5.73 ^Aa^	85.61 ± 4.88 ^ABab^	85.71 ± 4.88 ^ABa^

Note: Values are shown as means ± standard deviation; ^a,b,c^ means with different superscripts are significantly different (*p* < 0.05); ^A,B^ means with different superscripts are very significantly different (*p* < 0.01); body weight for six months (BW-6), body length for six months (BL-6), body height for six months (BH-6), chest circumference for six months (CC-6), and so on.

**Table 6:** Association analysis between haplotype combination and lambing number of Nanjiang Yellow goats.

Combined Haplotypes	Number	Primiparity	Multiparity
H2H3	21	1.52 ± 0.51	1.82 ± 0.26 ^Cc^
H3H3	23	1.74 ± 0.45	1.95 ± 0.15 ^BCb^
H1H2	18	1.67 ± 0.49	1.94 ± 0.24 ^BCb^
H1H3	47	1.57 ± 0.54	1.98 ± 0.21 ^Bb^
H2H2	34	1.65 ± 0.54	2.13 ± 0.17 ^Aa^

Note: Values are shown as means ± standard deviation; ^a,b,c^ means with different superscripts are significantly different (*p* < 0.05); ^A,B,C^ means with different superscripts are very significantly different (*p* < 0.01).

## Data Availability

The data from this study are exhibited in this manuscript and Supplementary Materials.

## Supplementary Materials

The following supporting information can be downloaded at: https://www.mdpi.com/article/10.3390/genes15030330/s1, Table S1. Transcription factors prediction. Table S2. The association between six SNPs and lambing performance in primiparous and multiparous Nanjiang Yellow goat populations. Table S3. RT-qPCR primers’ information. Table S4. Primers for exon validation. Table S5. Primers’ information of homologous recombination. Table S6. Information of site-directed mutagenesis primers. Table S7. Primers’ information of non-coding-region SNPs of *B4GALNT2*. Table S8. The primers information of enzyme digestion sites. Table S9. Primers’ information of enzyme digestion sites. Table S10. Basic data of growth, development, and reproductive performance of Nanjiang Yellow goats. Figure S1. Identification results. Pax7 (red) and MyHC (red) immunofluorescence staining were performed in MuSCs. Figure S2. Gel electrophoresis of genomic DNA samples. M. DL2000 DNA maker; (1–6) Genomic DNA of Nanjiang Yellow goats. Figure S3. Homology analysis between 6 SNPs in the *B4GALNT2* gene of goats and 42 other species. Figure S4. Phylogenetic tree of *B4GALNT2*.
