# Supplementary material for: An Identification of Functional Genetic Variants in B4GALNT2 Gene and Their Association with Growth Traits in Goats
Source: Genes (Basel). 2024 Mar 3;15(3):330. doi: 10.3390/genes15030330 (PMC10970026; doi:10.3390/genes15030330)
Supplement: Supplementary file 1 [file genes-15-00330-s001.zip › Figure S3. Homology analysis between 6 SNPs in t.docx]

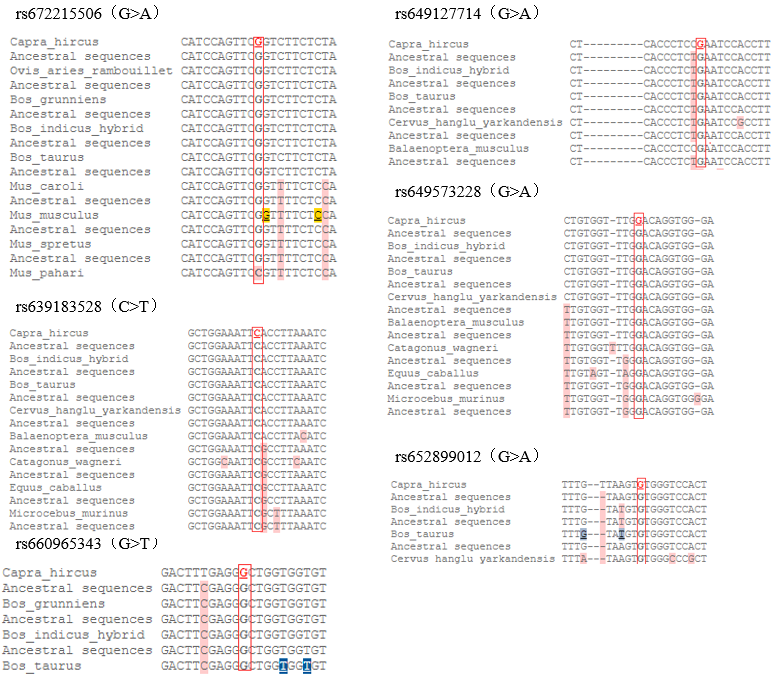


**Figure S3.** Homology analysis between 6 SNPs in the B4GALNT2 gene of goats and 42 other species (only parts are shown in the figure). rs672215506 was a synonymous mutation located in the exon, and the other SNPs were located in the non-coding region.
