# Supplementary material for: An Identification of Functional Genetic Variants in B4GALNT2 Gene and Their Association with Growth Traits in Goats
Source: Genes (Basel). 2024 Mar 3;15(3):330. doi: 10.3390/genes15030330 (PMC10970026; doi:10.3390/genes15030330)
Supplement: Supplementary file 1 [file genes-15-00330-s001.zip › Table S3-S9.docx]

Table S3. RT-qPCR primers information

| Gene | Primer sequence（5'–3'） | Tm（℃） | Product length（bp） |
| --- | --- | --- | --- |
| *B4GALNT2-Goat* | F: TCCGCTTTCCTGTTATGCC | 59.0 | 161 |
|  | R: AGCCACGATCACAGTCAAGTC |  |  |
| *GAPDH-*  *Goat* | F: GCAAGTTCCACGGCACAG | 59.0 | 249 |
|  | R: GGTTCACGCCCATCACAA |  |  |

Table S4. Primers for exon validation

| Locus | Primer sequence  （5'–3'） | Tm  （℃） | Product length（bp） |
| --- | --- | --- | --- |
| rs648256996  rs651860188 | F: TGGTTCTTGAAGTAGTGGAG  R: GAGTCCACAGTTCCGTTTA | 61.4 | 398 |
| rs650919960 | F: CCACGGGCTCATGTCTTAT  R: ATTCACAGCACCCATCCAA | 59.7 | 413 |
| rs670079545  rs658134198  rs652059999 | F: GAGTGGGAGTGCCTTTCTTCT  R: TGGATGGTGGCTGTGAGAA | 61.4 | 362 |
| rs670895166  rs659650872  rs643432049 | F: AAGGTGGGCACAATAGAAC  R: TCCTTGGGCTTTAGCACAT | 59.7 | 511 |
| rs672215506 | F:AAGTAAGAATGGCGGCAGAA  R:AACTGAGAAAGCTGCCAAGC | 61.1 | 370 |

Table S5. Primers Information of homologous recombination

| Gene | Primer sequence（5'–3'） | Tm（℃） | Product length（bp） |
| --- | --- | --- | --- |
| *B4GALNT2* | F:ctaccggactcagatctcgagATGACTTCGTTCGGCTCTAGATATC | 62.5 | 1521 |
|  | R:atggtggcgaccggtggatccTGTGGTACATTGGAGATGGTTCTT |  |  |

Table S6. Information of site-directed mutagenesis primers

| Gene | Primer sequence（5'–3'） | Tm（℃） | | Product length（bp） |
| --- | --- | --- | --- | --- |
| *B4GALNT2* | GAGAAGACcGAACTGGATGTGGTAGGTGGCAG | | 58.5 | 1521 |
|  | TCCAGTTCgGTCTTCTCTAGGACATCCACCAGC | |  |  |

Table S7. Primers information of non-coding region SNPs of *B4GALNT2*

| Locus | Primer sequence（5' – 3'） | Tm（℃） | Product length（bp） |
| --- | --- | --- | --- |
| rs660965343  （G>T） | F:AATGGCAATGGGTGGATATT  R:CACCCTAGGTCACCCTGAAA | 58.8 | 324 |
| rs649127714  （G>A） | F:TCGTCCATCCAGATTCAAC  R:GATTTAGAGGCAGGGCTTT | 62.0 | 254 |
| rs639183528  （C>T） | F:ACTGTCAGACGCAGCTTCAA  R:AGAACTGTCTGCCCCAAAGA | 59.0 | 802 |
| rs649573228  （G>A） | F:ACTGTCAGACGCAGCTTCAA  R:AGAACTGTCTGCCCCAAAGA | 59.0 | 802 |
| rs652899012  （G>A） | F:GTGACAAGACACCAACGA  R:TTGCCTGATGAGATTGACA | 63.1 | 543 |

Table S8. The primers information of enzyme digestion sites

| locus | Primer sequence（5'–3'） | Tm（℃） | Product length（bp） |
| --- | --- | --- | --- |
| rs660965343（G>T） | F:CGGGGTACCGCCTGCAAATACTCCCACTC | 55.0 | 629 |
|  | R:CCCAAGCTTCCTCCTGTCAACTGGTGTCAT |  |  |
| rs649127714  （G>A） | F:CGGGGTACCGGGCACAGGATTTAACTTGC | 55.0. | 572 |
|  | R:CCCAAGCTTTTCACCTCGCACTTCATCTG |  |  |
| rs639183528  （C>T） | F:CGGGGTACCCCTTTGCTTTCCGGTAGTGT | 55.0 | 440 |
|  | R:TCCCCCGGGTTTCTTCCCATGACGTTCC |  |  |
| rs649573228  （G>A） | F:CGGGGTACCTGGCAGTTTGAACCAGATA | 55.0 | 322 |
|  | R:TCCCCCGGGCAAGGGATTTGGGAATGTAA |  |  |
| rs652899012  （G>A） | F:CGGGGTACCGCATATAGAGCCAGAAACAC | 55.0 | 459 |
|  | R:TCCCCCGGGCCATTGGTGGACGAGACA |  |  |

Table S9. Primers of Mass-array genotyping

| locus | forward primer | reverse primer | extension primer |
| --- | --- | --- | --- |
| rs672215506 | ACGTTGGATGAGAGGAGATAGAACCAGCAG | ACGTTGGATGAGACCAAGATTGAGGTGCTG | GTGGATGTCCTAGAGAAGAC |
| rs660965343 | ACGTTGGATGGCTTAGGGACCCAGAAAAAC | ACGTTGGATGAGAAATAGAAGCCAGGGAGG | GGAGGAAGACTTTGAGG |
| rs649127714 | ACGTTGGATGAATGGTGCCATCAGACTGTG | ACGTTGGATGAGGAGGCCTCATGACTGAAG | CATGACTGAAGGTGGATT |
| rs639183528 | ACGTTGGATGCAGACATCGGCCATTTAGTG | ACGTTGGATGCTGATGGCAGTTTGAACCAG | gaagaATGCTTGCTGCTGGAAATT |
| rs649573228 | ACGTTGGATGCCAGAAACACAAGTTGGGAG | ACGTTGGATGTAGAGATGATACGAGGCCAG | TAAGCTTCCCACCTGT |
| rs652899012 | ACGTTGGATGACTGGCCCTTCTTTCGATTG | ACGTTGGATGACCCACCACCCTAAATCAAG | cCCCTAAATCAAGGATTTGTTAAGT |
