# Supplementary figures and images for: An Identification of Functional Genetic Variants in B4GALNT2 Gene and Their Association with Growth Traits in Goats
Source: Genes (Basel). 2024 Mar 3;15(3):330. doi: 10.3390/genes15030330 (PMC10970026; doi:10.3390/genes15030330)

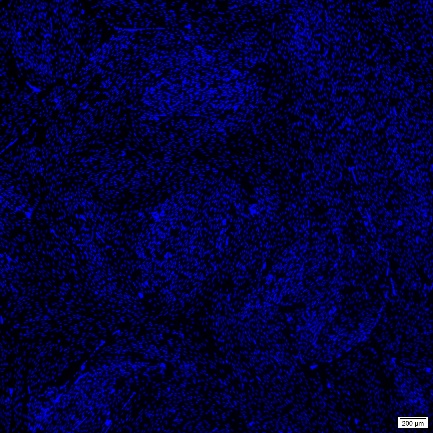

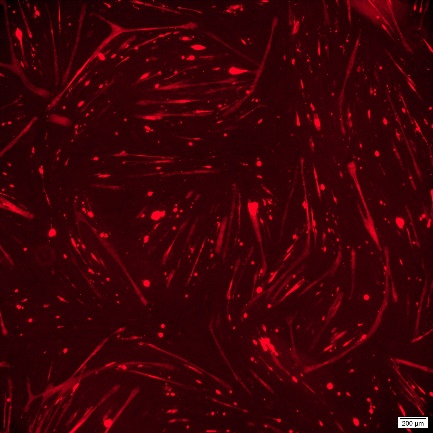

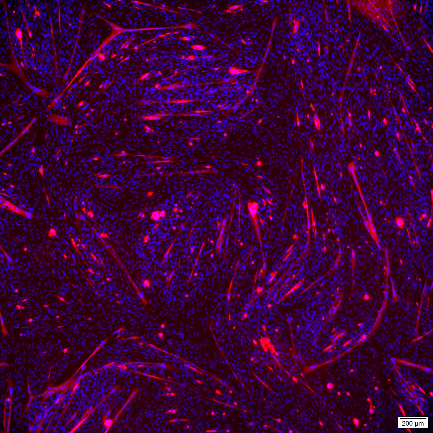

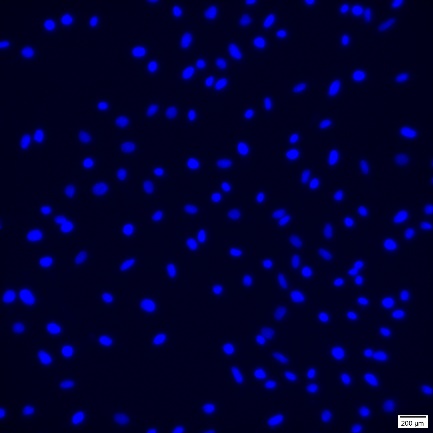

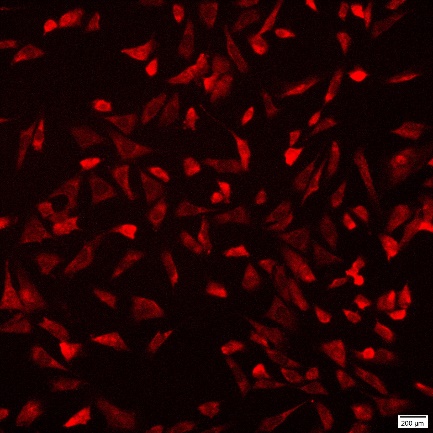

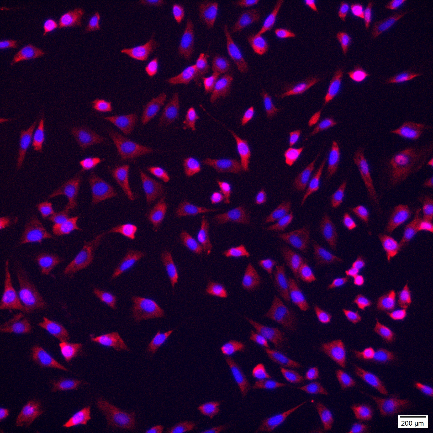


GM

DM

DAPI

Pax 7

Merge

Merge

DAPI

MYHC

Supplement: Supplementary file 1 [file genes-15-00330-s001.zip › Figure S1.The identification results.Pax7 (red) and MyHC (red) immunofluorescence staining were performed in MuSCs.docx]

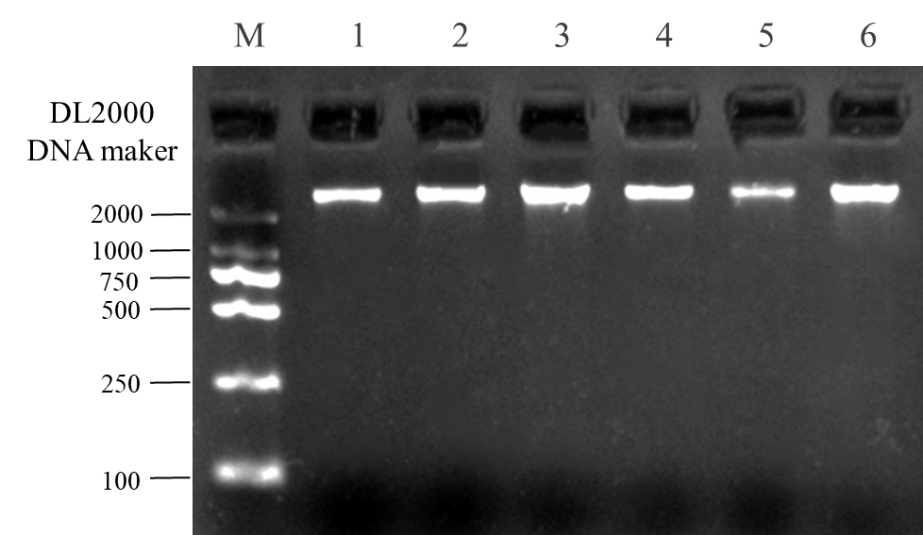


Note: M DL2000 DNA maker; 1-6. Genomic DNA of NanJiang Yellow goat.

Supplement: Supplementary file 1 [file genes-15-00330-s001.zip › Figure S2. Gel electrophoresis of genomic DNA samples.docx]

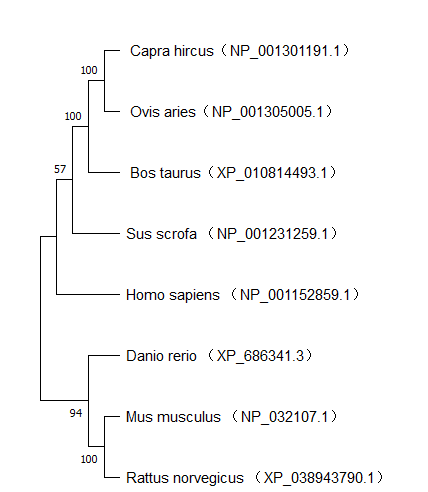


**Figure S4**. Phylogenetic tree of B4GALNT2

Supplement: Supplementary file 1 [file genes-15-00330-s001.zip › Figure S4. Phylogenetic tree of B4GALNT2.docx]
